# Comparative study of motor cortical excitability changes following anodal tDCS or high‐frequency tRNS in relation to stimulation duration

**DOI:** 10.14814/phy2.14595

**Published:** 2020-09-30

**Authors:** Jan Haeckert, Christoph Lasser, Benjamin Pross, Alkomiet Hasan, Wolfgang Strube

**Affiliations:** ^1^ Department of Psychiatry and Psychotherapy University Hospital Munich München Germany; ^2^ Department of Psychiatry, Psychotherapy and Psychosomatics, Bezirkskrankenhaus Augsburg University of Augsburg Augsburg Germany

**Keywords:** 1 mA intensity, anodal transcranial direct current stimulation, high‐frequency transcranial random noise stimulation, non‐invasive brain stimulation, stimulation duration

## Abstract

**Background:**

In this study, we investigate the capacity of two different non‐invasive brain stimulation (NIBS) techniques (anodal transcranial direct current stimulation (anodal tDCS) and high‐frequency transcranial random noise stimulation (hf‐tRNS)) regarding the relationship between stimulation duration and their efficacy in inducing long‐lasting changes in motor cortical excitability.

**Methods:**

Fifteen healthy subjects attended six experimental sessions (90 experiments in total) and underwent both anodal tDCS of 7, 13, and 20 min duration, as well as high‐frequency 1mA‐tRNS of 7, 13, and 20 min stimulation duration. Sessions were performed in a randomized order and subjects were blinded to the applied methods.

**Results:**

For anodal tDCS, no significant stable increases of motor cortical excitability were observed for either stimulation duration. In contrast, for hf ‐tRNS a stimulation duration of 7 min resulted in a significant increase of motor cortical excitability lasting from 20 to 60 min poststimulation. While an intermediate duration of 13 min hf‐tRNS failed to induce lasting changes in motor cortical excitability, a longer stimulation duration of 20 min hf‐tRNS led only to significant increases at 50 min poststimulation which did not outlast until 60 min poststimulation.

**Conclusion:**

Hf‐tRNS for a duration of 7 min induced robust increases of motor cortical excitability, suggesting an indirect proportional relationship between stimulation duration and efficacy. While hf‐tRNS appeared superior to anodal tDCS in this study, further systematic and randomized experiments are necessary to evaluate the generalizability of our observations and to address current intensity as a further modifiable contributor to the variability of transcranial brain stimulation.

## Graphical abstract

In the case of anodal tDCS, we did not observe stable increases of motor cortical excitability for either stimulation duration. By contrast, hf‐tRNS for a stimulation duration of 7 minutes resulted in a significant increase of motor cortical excitability lasting from 20 to 60 minutes post stimulation. In context of the ongoing debate regarding contributing factors to variability following non‐invasive transcranial brain stimulation, the findings of our experimental comparative study substantiate circumstantial yet novel evidence for a potential indirect proportional relationship between stimulation duration and efficacy in the case of hf‐tRNS.

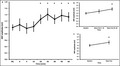

## 1 INTRODUCTION

Non‐invasive brain stimulation (NIBS) is largely viewed as a safe and effective approach to investigate neuronal functioning and neuroplasticity changes in the human brain. During the last decades, different stimulation protocols have been established which are viewed to induce excitability changes that outlast the stimulation interventions themselves. These effects have been either related to so‐called long‐term potentiation (LTP) like plasticity or long‐term depression (LTD) like plasticity (Paulus, 2011; Inukai et al., 2016; Dissanayaka et al., 2017) referring to respective increases or decreases in excitability of stimulated brain areas. In the majority of neurophysiological experiments that determined such long‐lasting after‐effects of NIBS, the human motor cortex (M1) has been used as a model system. There, it is possible to investigate induced cortical excitability changes by means of single‐pulse motor cortical transcranial magnetic stimulation (TMS) in combination with peripheral electromyography (EMG). Paired‐pulse TMS paradigms like short‐intracortical inhibition (SICI) or short intracortical facilitation (SICF) are used to assess intracortical inhibitory and excitatory synaptic modulations of M1.

It is important to note, that tDCS after‐effects have been shown to be subject to relevant inter‐subject variability (Wiethoff et al., 2014; Strube et al., 2016; Guerra et al., 2020). A number of studies showed, that the response to NIBS protocols is rather variable in healthy humans and there is a substantial portion of subjects considered as so‐called non‐responders (i.e., subjects showing a different tDCS after effect than expected on the group level by foregoing findings). Apart from that, several factors such as age, gender, handness, skull confirmation, skin condition, arousal, sleep deprivation prior to the stimulation, the state of the motor system activation, as well as hormones and their cyclic fluctuations lead to intra‐ und inter‐subject variability (Ridding and Ziemann, 2010; Guerra et al., 2020). The increasing recognition of the high variability in the reported effects of tDCS, even when using the same stimulation parameters, makes the effect of tDCS anything but predictable (Li et al., 2015). Furthermore, the factors affecting the biological response to electrical current are highly important, yet in the view of some experts underappreciated (Li et al., 2015). Meanwhile, it was also reported, that even after a sham stimulation significant changes in MEP amplitude compared to baseline could be observed (Kortuem et al., 2019), adding further possible impacting factors to a growing field of interest in NIBS efficacy.

One meta‐analysis concluded that across studies anodal tDCS (a‐tDCS) increases and cathodal tDCS (c‐tDCS) decreases motor cortical excitability (Dissanayaka et al., 2017). Regarding the postulated mechanisms by which tDCS induces neuroplastic changes in motor cortical excitability, it is assumed that tDCS induces sustained elevations (in the case of anodal tDCS) or decreases (in the case of cathodal tDCS) in neural cell membrane potentials (Nitsche et al., 2009). Further, complementary findings from neurophysiological human motor cortex studies, as well as experiments in rodent models indicate, that changes in glutamatergic neurotransmission and especially N‐methyl‐D‐aspartate receptors (NMDA‐R) might mediate—at least to some extend—direct current stimulation‐induced plasticity (Stagg et al., 2018). This potential association is supported by interventional studies which demonstrated, that pharmacological blockade of NMDA receptors prevents or mitigates tDCS‐induced excitability alterations, both for anodal and cathodal tDCS, whereas NMDA receptor agonists can enhance anodal tDCS‐induced excitability changes (Nitsche et al., 2003; Paulus, 2011). In 2008, Terney et al. reported on a new stimulation paradigm with tRNS, which was demonstrated also capable of inducing LTP‐like plasticity after‐effects in the stimulated motor cortex. Here, tRNS was shown to increase motor cortical excitability in 17 healthy subjects, while sham stimulation failed to do so (Terney et al., 2008). Where tDCS uses constant direct currents delivered for several minutes, tRNS uses random levels of currents alternating in amplitude and frequency using a spectrum ranging from 0.1 Hz to 640 Hz. Based on subsequent neurophysiological experiments it was shown, that higher frequency bands between 100 and 640 Hz, so‐called high‐frequency tRNS (hf‐tRNS), could be more effective in generating increases in poststimulation motor cortical excitability (Paulus, 2011), compared to frequencies below 100 Hz (Terney et al., 2008) or above 640 Hz (Moret et al., 2019). To this extend, Moret et al. showed, moreover, that current levels of 1.5 mA in the case of tRNS, for 10 min resulted in significant and stable increases of motor cortical excitability in 11 healthy participants only, when the full‐band of frequencies was applied (100–700 Hz), while more restricted spectra (100–400 Hz, 400–700 Hz) were unable to induce stable after‐effects (Moret et al., 2019). As with tDCS, the exact mechanisms of how tRNS induces the observed long lasting after‐effects are yet unclear. However, it has been proposed that tRNS induces the temporal summation of small depolarizing currents, which might interact with thereby engaged neurons (Moret et al., 2019; Pavan et al., 2019). Another possible explanation for tRNS after‐effects could be, that it would promote the activity of sodium channels (Terney et al., 2008). Using a comparative approach for both paradigms, Inukai et al. later reported on 10 min of 1 mA tRNS, which resulted in increased motor cortical excitability in 15 healthy subjects in a significantly more stable way, than anodal tDCS applied with the same stimulation parameters (1 mA, 10 min) (Inukai et al., 2016). Compared to tDCS, the novel paradigm of tRNS was first considered as a possible means of overcoming or identifying causes of the issue of variability. Finally, a comparative study investigating the different stimulation methods of transcranial alternating current stimulation (tACS), intermittent theta burst stimulation (iTBS), tDCS, and tRNS, and aiming to induce LTP‐like motor cortical excitability changes showed, that tRNS (1 mA, 10 min) resulted in the most distinct and significantly long‐lasting MEP amplitude increases compared to sham stimulation (Inukai et al., 2016). However, as indicated above, other studies have shown pronounced inter‐individual as well as intra‐individual variability with respect to the efficacy of NIBS to induce the after‐effects expected from landmark studies (Pellegrini et al., 2018a,b; Guerra et al., 2020). Since several biological and methodological factors have been identified or proposed to cause fragility of the desired after‐effects, following NIBS studies aiming to identify controllable contributory factors to variability, could prove relevant for the further development of these techniques (Cirillo et al., 2017).

In this regard, some unalterable biological factors previously identified as potential causes of NIBS variability consist of age, gender, genetic polymorphisms, individual brain anatomy, and functional brain engagement (Ridding and Ziemann, 2010). In contrast, other factors, such as exercise prior to brain stimulation or the time of the day when NIBS is delivered, are considered influenceable biological contributors to variability (Huang et al., 2017; Pellegrini et al., 2018a,b). In addition to biological contributors, several methodological parameters have been identified, that are viewed to largely contribute to NIBS efficacy and are at the same time modifiable. These consist of the stimulation protocols per se and the stimulation parameters, such as the applied current direction (anodal versus. cathodal), the intensity or level of the applied current (e.g., 1 mA versus. 2 mA), pulse configuration, and others, such as frequency bands, in the case of hf‐tRNS (Huang et al., 2017). In this context, Nitsche and Paulus, (2000) showed for tDCS, that the duration length of the applied stimulation necessary to induce after‐effects must outlast 3 min with a current intensity of at least 0.6 mA). Initially, these findings led to the consideration, that a linear association was hypothesized, where stimulation duration would proportionally affect how long the evoked after‐effects would last. However, consecutive experiments demonstrated that there may be an upper limit for sustaining the excitatory after‐effects resulting from anodal tDCS, as a stimulation of 26 min resulted in LTD like the inhibition of cortical excitability (Monte‐Silva et al., 2013). Reviewing the evidence regarding experiments applying different stimulation durations, the meta‐analysis of Dissanayaka and colleagues revealed that for anodal tDCS larger after‐effects were induced with current intensities of < 1mA and with durations of > 10 min, than following anodal tDCS of higher intensities and with durations of either < 10 min or > 10 min (Dissanayaka et al., 2017). Based on the original and replicated findings for anodal tDCS we first selected a stimulation duration of 13 min, as we expected (on the group level) relatively robust facilitatory changes in MEP magnitudes following this specific duration (Nitsche and Paulus, 2001). Based on work by the same group and in the context of foregoing hf‐tRNS experiments (see respective section below) we next chose a shorter stimulation duration of 7 min, which we regarded as a sham condition for tDCS and an intermediate duration for hf‐tRNS. Finally, as most therapeutic applications for tDCS nowadays employ a longer stimulation duration of 20 min we included this specific duration additionally into our experimental setup. For tRNS, Chaieb et al. showed that the stimulation duration in the case of 1mA tRNS needed to outlast 5 min to induce a long‐lasting excitability‐enhancement (Chaieb et al., 2011). However, due to yet limited data regarding tRNS, with most studies performed with a duration of 5 min and 10 min, the optimal combination of duration, frequency bands, and intensity has yet not been clarified. Further, systematic comparisons regarding the efficacy of anodal tDCS relative to tRNS regarding increases of motor cortical excitability in relation to specific simulation durations are yet lacking. Hence, as stimulation duration poses one of the potential contributors of variable efficacy and is at the same modifiable, the aim of our study was to investigate the after‐effects on motor cortical excitability of anodal tDCS compared to high‐frequency tRNS using three different stimulation durations (7, 13 or 20 min). Our hypothesis was that (a) there would be an optimal stimulation duration related to inducing robust after‐effects and (b) that high‐frequency tRNS would be more effective than tDCS in inducing lasting after‐effects.

## 2 METHODS

### 2.1 Subjects

Fifteen healthy subjects participated in this study after giving informed consent. None of the subjects had a history of neurological or mental illness or had metal brain implants, nor had a history of alcohol or drug abuse and none were taking any neuroactive medication. The study protocol, which is in accordance with the Declaration of Helsinki, was approved by the Ethics Committee of the Medical Faculty of the Ludwig Maximilian's University of Munich (reference number: 54–14).

### 2.2 Design

All 15 subjects attended six experimental sessions separated by at least one day and underwent both anodal tDCS of 7, 13 or 20 min and high‐frequency tRNS of 7, 13 or 20 min (resulting in six experimental sessions per subject). Stimulation intensity was set to 1 mA for all experiments as detailed below. Stimulation sessions for a given subject were performed during the same time of the day to mitigate the influence of circadian factors (Cirillo et al., 2017). Sessions were performed in a randomized order (randomization list created by https://www.random.org/lists/) and subjects were blinded to the applied method.

### 2.3 Experimental procedures

**Figure 1:**
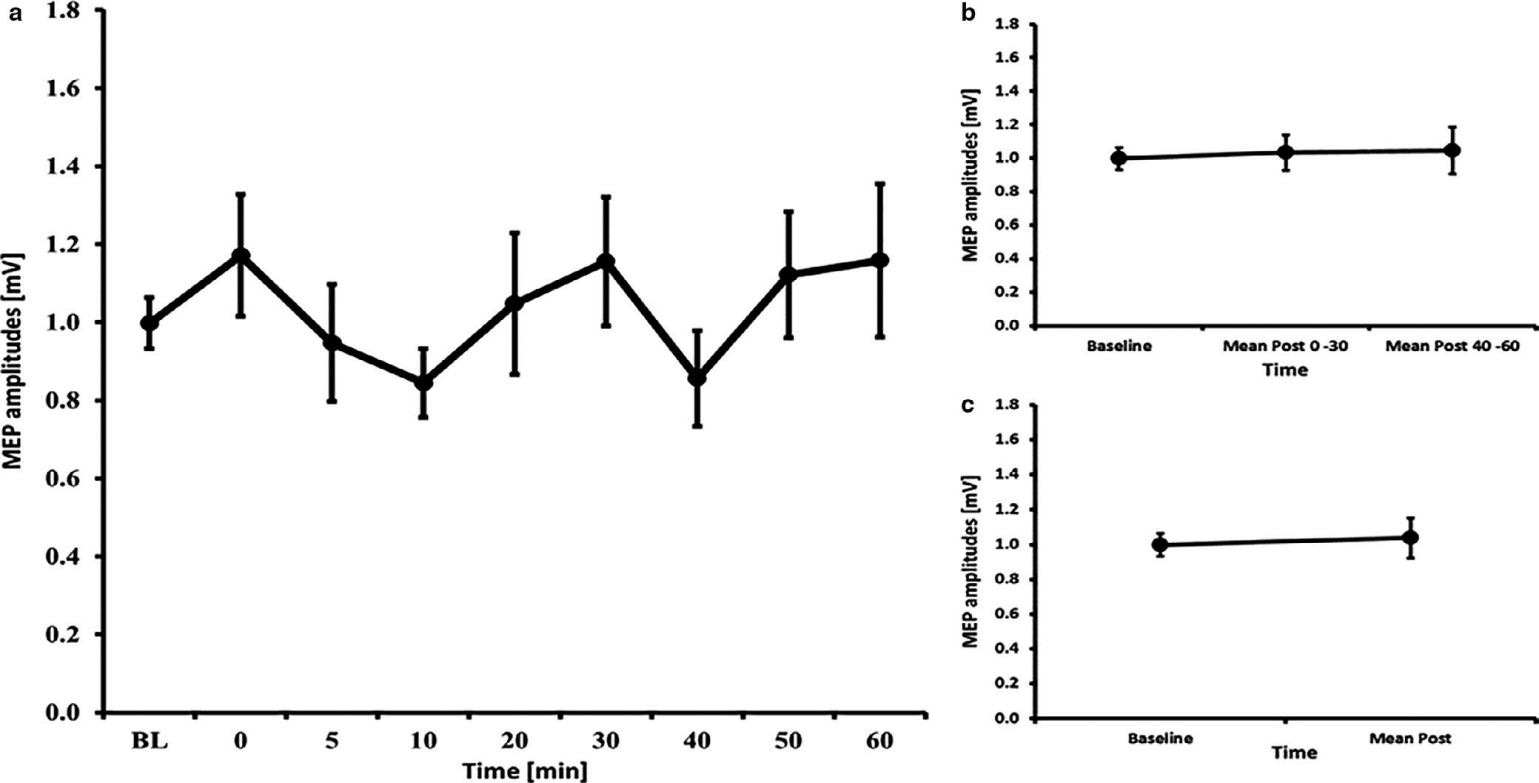
MEP courses for the 7 minutes anodal tDCS experiments A) course of all time bins B) course of the early and late epoch time bins C) course of the post mean MEP values. Error bars refer to the standard error of the mean.

During all experiments, participants sat in a comfortable chair with their head and arms at rest. As detailed elsewhere (Campana et al., 2019), we recorded electromyography activity (EMG) via surface electrodes on the right first dorsal interosseus muscle (FDI). Raw signals were amplified and bandpass‐filtered (2 Hz to 3 kHz range) using a Digitimer D‐360 amplifier setup (Digitimer Ltd, UK) and digitized at 5 kHz using a 1,401 data acquisition interface (Cambridge Electronic Design Ltd., Cambridge UK) controlled by Signal Software (Version 5, Cambridge Electronic design, Cambridge UK). At the end of the study, all data were analyzed off‐line. During the experiments, complete muscle relaxation was controlled by visual feedback of EMG activity. As outlined elsewhere (Hasan et al., 2012), motor cortex TMS to access cortical excitability was performed with a standard figure‐of‐eight coil (70 mm, The Magstim Company Ltd, UK) connected to a Magstim Bistim^2^ stimulator (The Magstim Company Ltd, UK). In all experiments, the coil was held tangentially to the skull above the left primary motor cortex (M1), with the handle pointing in a dorsolateral direction at a 45° angle from the midsagittal line leading to a posterior–anterior directed current (Di Lazzaro et al., 1998). Optimal coil positioning was defined over the region where most stable MEP were evoked and marked with a pen on the skin for further tDCS/tRNS and TMS stimulation. After completing the baseline parameter assessments, the anodal saline‐soaked sponge electrode (35 cm^2^) was placed in the middle of the marked position and fixed with a rubber band. The cathodal electrode was located over the right forehead, with the lower margin of the cathodal electrode beginning at the eyebrows also fixated with a rubber band (see Figure 1 in (Nitsche et al., 2008)). After tDCS/tRNS stimulation, the sponge electrodes were removed and the TMS coil repositioned at the pre‐defined optimal coil position for poststimulation measurements.

### 2.4 Transcranial direct current stimulation (tDCS)

Anodal tDCS was applied according to previously published protocols (Di Lazzaro et al., 1998; Nitsche and Paulus, 2000). In short, constant current was administered through a pair of saline‐soaked sponge electrodes (35 cm^2^). The anode was placed over the representational area of the motor cortex of the left hemisphere and the cathode was placed over the contralateral orbit. Dependent on the stimulation protocol 1 mA current was applied for 7, 13 or 20 min, respectively, with a fade in and fade out time of 15 s.

### 2.5 High‐frequency transcranial random noise stimulation (hf‐tRNS)

High‐frequency tRNS was applied according to previously published protocols (Terney et al., 2008). In short, a random level of current was generated for every sample (sampling rate: 1,280 samples/s with random numbers normally distributed and a probability density function following a bell‐shaped curve) (Terney et al., 2008). As for tDCS, the stimulation electrode was fixed at the motor cortex position of the left hemisphere, the reference electrode was placed over the contralateral orbit. Dependent on the stimulation protocol, hf‐tRNS was applied for 7, 13 or 20 min, respectively, with a current strength of 1 mA (offset 0 mA) (Terney et al., 2008) with a fade in and fade out time of 15 s and an offset set to zero (Terney et al., 2008).

### 2.6 Baseline excitability and monitoring of excitability changes

Before anodal tDCS or hf‐tRNS were applied, baseline parameters of motor cortical excitability were assessed at each experimental session. Single‐pulse TMS measurements included the intensity to evoke MEP of approximately 1mV (peak‐to‐peak amplitude (S1mV) and resting motor threshold (RMT). Single‐pulse MEP measurements using S1mV intensity were obtained both at baseline (40 stimuli) and following stimulation with tDCS or hf‐tRNS using single‐pulse MEP measurements (20 stimuli each) at predefined time intervals of 0, 5, 10, 20, 30, 40, 50, and 60 min to monitor after‐effects using S1mV. Additionally and in order to explore potential modulations of excitability parameters, input and output (I/O) curves were measured at baseline and after 20 min using seven stimuli with intensities of 90%, 110%, and 130% of RMT. These were applied in increasing order at 0.2Hz intervals with 10 s break between each intensity. Furthermore, additional explorative short‐latency intracortical inhibition and facilitation (SICI/ICF) parameters were recorded at baseline and after the recording of the 10 min time bin with a standardized paired‐pulse protocol (S1: 80% RMT, S2: S1mV; interstimulus intervals (ISI): 2, 3, 7, 9, and 12 ms) (Kujirai et al., 1993). The testpulse was applied 15 times, and all paired‐pulses were applied 10 times in a randomized order at 0.2 Hz. Due to the relatively high number of MEP measurements and due to their better comparability with foregoing experimental studies, we focused on I/O and SICI/ICF and refrained from performing SICF measurements in addition to I/O and SICI/ICF.

### 2.7 Statistical methods

Necessary sample sizes were calculated based on a power analysis using G*Power 3.1.9.2 (Faul et al., 2007). For an RM‐ANOVA approach with a 9‐level within‐subject factor (TIMECOURSE), an estimated moderate effect size of f = 0.25, α = 0.05, and a power of 1‐β = 0.80 with a correlation among measures of 0.5, this analysis obtained a necessary sample size of 15 subjects per experiment (defined as one group). This sample size is within the range of previous studies (Terney et al., 2008; Inukai et al., 2016; Strube et al., 2016).

SPSS 25 for Windows (IBM, Armonk, NY, USA) was used for all analyses and the level of significance was defined as α = 0.05. RMT, S1mV, and MEP amplitudes at baseline were compared using an RM‐ANOVA with the 6‐level within‐subject factor EXPERIMENT to test for differences across experimental sessions. After‐effects were analyzed employing an overall RM‐ANOVA with the within‐subject factor “TIMECOURSE” (Baseline, 1 min, 5 min, 10 min, 20 min, 30 min, 40 min, 50 min, and 60 min) and the within‐subject factor “DURATION” (7 min, 13 min, 20 min). Based on the aforementioned power‐calculation and on our consideration, that the different selected durations would result in divergent yet variable after‐effects (see also introduction section for further details on variability), we further computed explorative RM‐ANOVAs only employing the within‐subject factor “TIMECOURSE” (Baseline, 1 min, 5 min, 10 min, 20 min, 30 min, 40 min, 50 min, and 60 min) where computed for each duration separately. Next, we subdivided the post‐stimulation periods into “early” MEP (average of 1 min, 5 min, 10 min, 20 min, and 30 min) and “late” MEP magnitudes (average of 40 min, 50 min, and 60 min) to further investigate if the induced after‐effects evolve more in the early or late phase after the stimulation. Additionally, we calculated “mean‐post” MEP (the average of all post‐stimulation time bins) and computed paired‐samples *t*‐tests to compare baseline and average poststimulation MEP. in the case of significant main effects, LSD tests (estimated marginal means) were performed to pairwise compare MEP amplitudes at different time bins to baseline. For I/O curves, RM‐ANOVAs with the within‐subject factors “TIME” (pre and postintervention) and “INTENSITY” (90%, 110%, 130%) were conducted. For paired‐pulse measures (SICI, ICF), RM‐ANOVAs with the within‐subject factors “TIME” (pre and postintervention) and “ISI” (testpulse and 2, 3, 7, 9, and 12 ms) were conducted. Mauchly's test of sphericity was used to test the assumption of sphericity and, if significant, we applied the Greenhouse–Geisser correction. Data in the manuscript and tables show means values ± standard deviation (*SD*) and figure error bars refer to the standard error of the mean (*SEM*).

## 3 RESULTS

### 3.1 Baseline characteristics

Fifteen healthy subjects (10 female, all right‐handed, mean age: 23.93 ± 2.96) participated in a total of 90 experimental sessions. RMT (*F*_(5, 70)_ = 0.586, *p* = .710), S1mV (*F*_(5, 70_) = 0.501, *p* = .775) and baseline MEP amplitudes (*F*_(2.84, 39.69_) = 1.052, *p* = .378) did not differ across all six experiments (see Table 1).

**Table 1:** MEP, RMT, and SI1mV values in all experiments expressed as mean values ± standard deviation

	7 tDCS	13 tDCS	20 tDCS	7 hf‐tRNS	13 hf‐tRNS	20 hf‐tRNS	*p*
**Baseline‐MEP [mV]**	0.997 ± 0.255	1.132 ± 0.332	0.978 ± 0.197	0.999 ± 0.328	1.125 ± 0.345	1.179 ± 0.537	0.378
**RMT [%]**	31.47 ± 7.090	32.00 ± 7.051	31.07 ± 5.298	30.80 ± 6.281	30.73 ± 5.873	32.07 ± 5.418	0.710
**SI1mV [%]**	39.93 ± 7.986	39.00 ± 7.964	38.87 ± 7.170	39.20 ± 7.599	39.13 ± 7.070	38.53 ± 7.060	0.775

Note

mV: millivolt; %: percentage of stimulator output. *p*: p value from RM‐ANOVA (for all details see main text).

### 3.2 Overall effects

In the case of anodal tDCS, the overall RM‐ANOVA for both factors (“DURATION” and “TIMECOURSE”; 3 × 9) obtained no significant main effects of “DURATION” (F_1.2,16.8_ = 1.02, *p* = .343) nor of “TIMECOURSE” (F_4.0,55.6_ = 1.49, *p* = .218) as well as no significant “DURATION × TIMECOURSE” interaction (F_5.2,72.9_ = 0.77, *p* = .582). In contrast, for hf‐tRNS the overall RM‐ANOVA for both factors (“DURATION” and “TIMECOURSE”; 3 × 9) revealed a significant main effect of “TIMECOURSE” (F_8,104_ = 3.35, *p* = .002), while no significant effects for “DURATION” (F_2,26_ = 0.17, *p* = .842) and no significant “DURATION × TIMECOURSE” interaction (F_16,208_ = 1.27, *p* = .218) were observed. Since, following our hypotheses, we considered the selected stimulation durations (7 min, 13 min, and 20 min) as separate conditions in experimental terms and expected divergent after‐effects following these durations, we then conducted subsequent explorative analyses repeating RM‐ANOVAS for each of the selected durations.

### 3.3 tDCS – 7 minutes

The RM‐ANOVA for all time bins revealed no significant effect of TIMECOURSE (*F*_(3.08, 43.18)_ = 1.430, *p* = .247). The RM‐ANOVA subdivided into early and late epochs revealed no significant effect of TIMECOURSE (*F*_(2, 28)_ = 0.170, *p* = .844) and paired‐samples *t*‐tests comparing baseline to mean post‐MEP values also revealed no significant differences (t(14)=0.49, *p* = .633) (see Figure 1). The RM‐ANOVA for the I/O curves revealed a significant effect of INTENSITY (*F*_(1.19, 16.69)_ = 42.049, *p* < .001), but no effects of TIME (*F*_(1, 14)_ < 0.001, *p* = .999) or INTENSITY X TIME interaction (*F*_(2, 28)_ = 0.590, *p* = .561) (see Table 2). The RM‐ANOVA for SICI/ICF (conducted on 12 subjects because 3 subjects had missing data) showed a main effect of ISI (*F*_(5, 60)_ = 21.317, *p* = .001), but no main effect of TIME (*F*_(1, 12)_ = 0.930, *p* = .354) or ISI x TIMECOURSE interaction (F_2.51, 30.10)_ = 1.186, *p* = .327) (see Table 3).

**Table 2:** I–O curve values in all experiments in mV; %: percentage of stimulator output

	Mean	SD	Mean	SD	Mean	SD
**prae Stimulation**	**7 tDCS**		**13 tDCS**		**20 tDCS**	
90% RMT	0.045	0.038	0.067	0.041	0.036	0.023
110% RMT	0.418	0.385	0.573	0.421	0.400	0.412
130% RMT	1.482	0.864	1.522	0.708	1.492	1.151
**post Stimulation**						
90% RMT	0.048	0.040	0.069	0.068	0.049	0.032
110% RMT	0.353	0.364	0.412	0.284	0.444	0.366
130% RMT	1.545	0.878	2.058	1.347	1.677	1.294
**prae Stimulation**	**7 tRNS**		**13 tRNS**		**20 tRNS**	
90% RMT	0.044	0.039	0.051	0.040	0.049	0.033
110% RMT	0.295	0.268	0.342	0.328	0.433	0.304
130% RMT	1.316	0.660	1.478	0.765	1.775	0.936
**post Stimulation**						
90% RMT	0.054	0.048	0.055	0.048	0.058	0.094
110% RMT	0.324	0.250	0.487	0.470	0.600	0.523
130% RMT	1.474	0.652	1.653	1.004	2.156	1.819

**Table 3:** SICI/ICF values in all experiments in mV

	Mean	*SD*	Mean	*SD*	Mean	*SD*
**prae Stimulation**	**7 tDCS**		**13 tDCS**		**20 tDCS**	
Testpulse	1.049	0.344	0.963	0.441	1.089	0.388
2 ms	0.443	0.310	0.415	0.326	0.602	0.530
3 ms	0.335	0.200	0.375	0.368	0.381	0.281
7 ms	1.189	0.596	1.482	0.771	1.381	0.738
9 ms	1.469	0.560	1.661	0.627	1.582	0.625
12 ms	1.432	0.758	2.004	0.942	1.795	0.700
**post Stimulation**						
Testpulse	0.909	0.502	1.316	0.618	1.337	0.709
2 ms	0.595	0.626	0.532	0.301	0.728	0.699
3 ms	0.348	0.313	0.430	0.414	0.462	0.453
7 ms	1.414	0.663	1.762	1.155	1.604	0.960
9 ms	1.474	0.737	2.046	1.087	2.025	1.225
12 ms	1.731	0.990	2.118	1.029	1.873	0.989
**prae Stimulation**	**7 tRNS**		**13 tRNS**		**20 tRNS**	
Testpulse	1.001	0.377	1.085	0.493	1.020	0.461
2 ms	0.429	0.395	0.546	0.513	0.528	0.432
3 ms	0.444	0.318	0.391	0.293	0.361	0.296
7 ms	1.475	0.759	1.303	0.555	1.226	0.785
9 ms	1.688	0.695	1.773	0.636	1.480	0.803
12 ms	1.849	0.836	1.632	0.584	1.624	0.782
**postStimulation**						
Testpulse	1.095	0.433	1.353	0.659	1.110	0.668
2 ms	0.563	0.511	0.534	0.404	0.505	0.409
3 ms	0.489	0.393	0.505	0.378	0.357	0.318
7 ms	1.447	0.846	1.474	0.876	1.347	1.142
9 ms	1.799	0.914	1.625	0.834	1.383	0.757
12 ms	2.090	1.039	1.906	0.944	1.714	0.984

### 3.4 tDCS – 13 minutes

**Figure 2:**
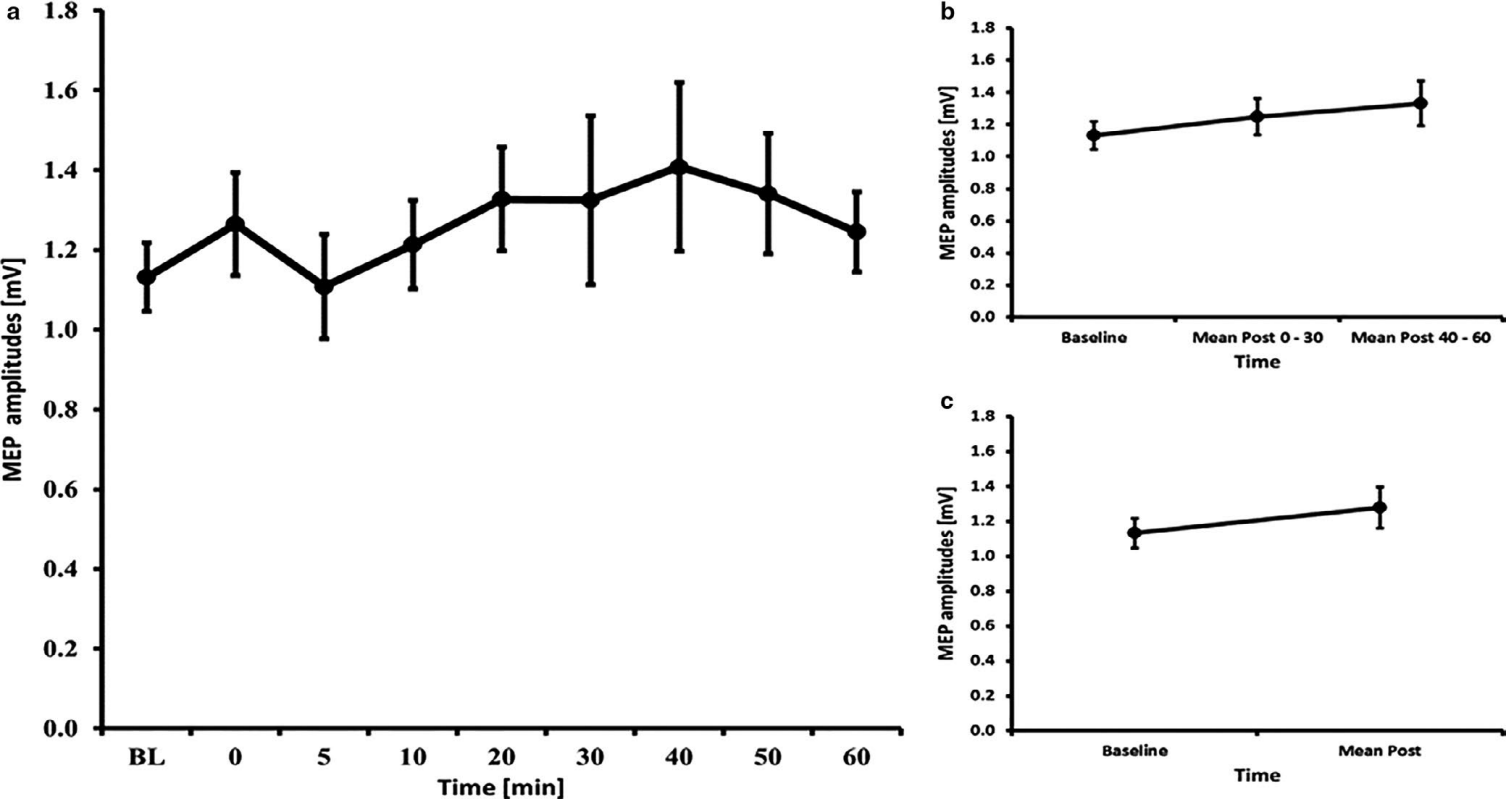
MEP courses for the 13 minutes anodal tDCS experiments A) course of all time bins B) course of the early and late epoch time bins C) course of the post mean MEP values. Error bars refer to the standard error of the mean.

The RM‐ANOVA for all time bins revealed no significant effect of TIMECOURSE (*F*_(3.51, 49.15)_ = 0.952, *p* = .434). The RM‐ANOVA subdivided into early and late epochs revealed no significant effect of TIMECOURSE (*F*_(2, 28)_ = 1.879, *p* = .172) and paired‐samples *t*‐tests comparing baseline to mean post‐MEP values also revealed no significant differences (t(14)=1.33, *p* = .204) (see Figure 2). The RM‐ANOVA for the I/O curves revealed a significant effect of INTENSITY (*F*_(1.19, 16.71)_ = 54.952, *p* < .001), but no effects of TIME (*F*_(1, 14)_ = 0.859, *p* = .370) or INTENSITY X TIME interaction (*F*_(1.15, 16.14)_ = 3.062, *p* = .095) (see Table 2). The RM‐ANOVA for SICI/ICF (conducted on 14 subjects because 1 subject had missing data) showed a main effect of ISI (*F*_(5; 65)_ = 28.494, *p* < .001), but no main effect of TIME (*F*_(1, 13)_ = 2.740, *p* = .122) and ISI X TIME interaction (F_2.54, 33.03)_ = 0.985, *p* = .401) (see Table 3).

### 3.5 tDCS – 20 minutes

**Figure 3:**
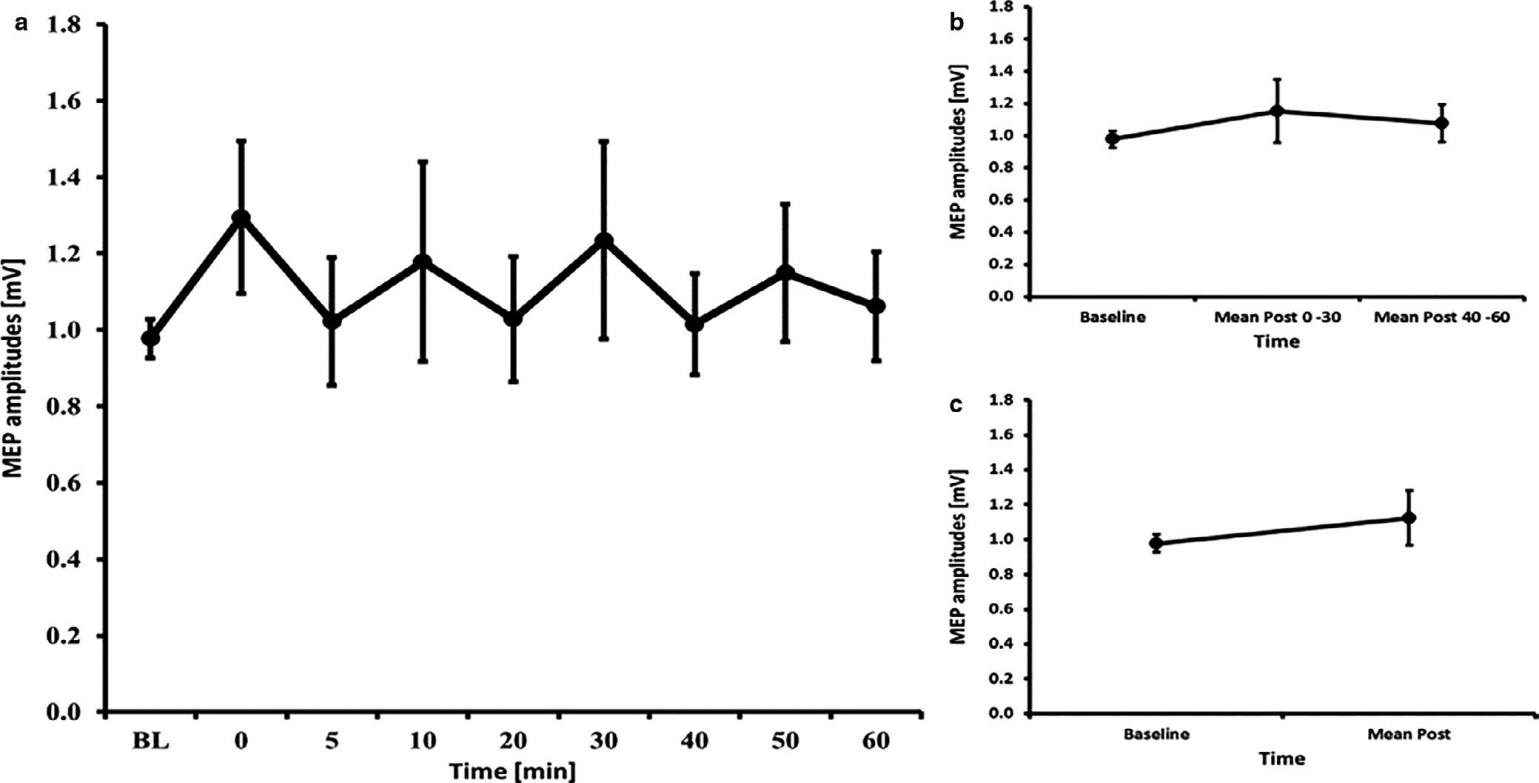
MEP courses for the 20 minutes anodal tDCS experiments A) course of all time bins B) course of the early and late epoch time bins C) course of the post mean MEP values. Error bars refer to the standard error of the mean.

The RM‐ANOVA for all time bins revealed no significant effect of TIMECOURSE (*F*_(2.68, 37.48)_ = 0.780, *p* = .499). The RM‐ANOVA subdivided into early and late epochs revealed no significant effect of TIMECOURSE (*F*_(2, 28)_ = 0.626, *p* = .542) and paired‐samples *t*‐tests comparing baseline to mean post‐MEP values also revealed no significant differences (t(14) = 0.96, *p* = .355) (see Figure 3). The RM‐ANOVA for the I/O curves revealed a significant effect of INTENSITY (*F*_(1.07, 15.04)_ = 27.044, *p* < .001), but no effects of TIME (*F*_(1, 14)_ = 0.866, *p* = .368)or INTENSITY X TIME interaction (*F*_(1.13, 15.75)_ = 0.450, *p* = .535) (see Table 2). The RM‐ANOVA for SICI/ICF (conducted on 14 subjects because 1 subject had missing data) showed a main effect of ISI (*F*_(2.74, 35.56)_ = 35.002, *p* < .001), but no effects of TIME (*F*_(1, 13)_ = 2.694, *p* = .125) or ISI X TIME interaction (*F*_(2.88, 37.44)_ = 1.023, *p* = .391) (see Table 3).

### 3.6 hf‐tRNS – 7 minutes

**Figure 4:**
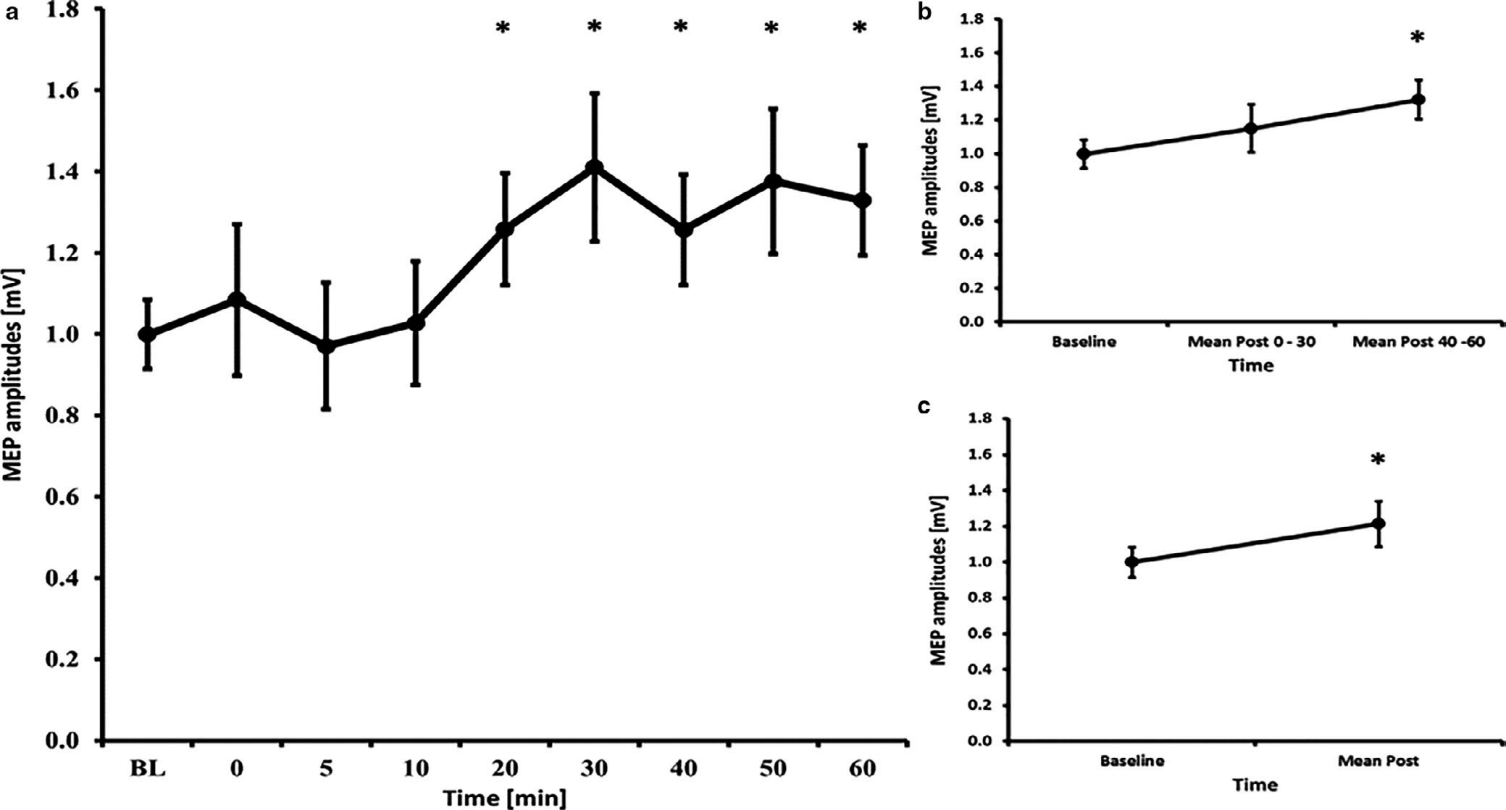
MEP courses for the 7 minutes high frequency tRNS experiments A) course of all time bins B) course of the early and late epoch time bins C) course of the post mean MEP values. Error bars refer to the standard error of the mean (*p<0.05).

The RM‐ANOVA for all time bins revealed a main effect of TIMECOURSE (*F*_(8, 112)_ = 2.925, *p* = .005). LSD tests revealed significant higher MEP amplitudes compared to baseline at 20 min (*p* = .029), 30 min (*p* = .025), 40 min (*p* = .023), 50 min (*p* = .046) and 60 min (*p* = .008) after Stimulation (all other time bins *p* ≥ .565). Also the RM‐ANOVA subdivided into early and late epochs revealed a significant effect of TIMECOURSE (*F*_(2, 28)_ = 6.465, *p* = .005) and LSD tests revealed increased MEP amplitudes for the later epochs (*p* = .003), but not for the early epochs (*p* = .162). Further, the paired‐samples *t*‐test comparing baseline to mean post‐MEP values also revealed a significant effect (t(14)=2.39, *p* = .032) (see Figure 4). The RM‐ANOVA for the I/O curves revealed a significant effect of INTENSITY (*F*_(1.22, 17.08)_ = 69.422, *p* < .001), but no effects of TIME (*F*_(1, 14)_ = 0.822, *p* = .380) or INTENSITY X TIME interaction (*F*_(1.25, 17.51)_ = 0.542, *p* = .510) (see Table 2). The RM‐ANOVA for SICI/ICF showed a main effect of ISI (*F*_(2.55, 35.71)_ = 44.485, *p* < .001), but no effects of TIME (*F*_(1, 14)_ = 1.036, *p* = .326) or ISI X TIME interaction (*F*_(3.13, 43.86)_ = 0.650, *p* = .594) (see Table 3).

### 3.7 hf‐tRNS – 13 minutes

**Figure 5:**
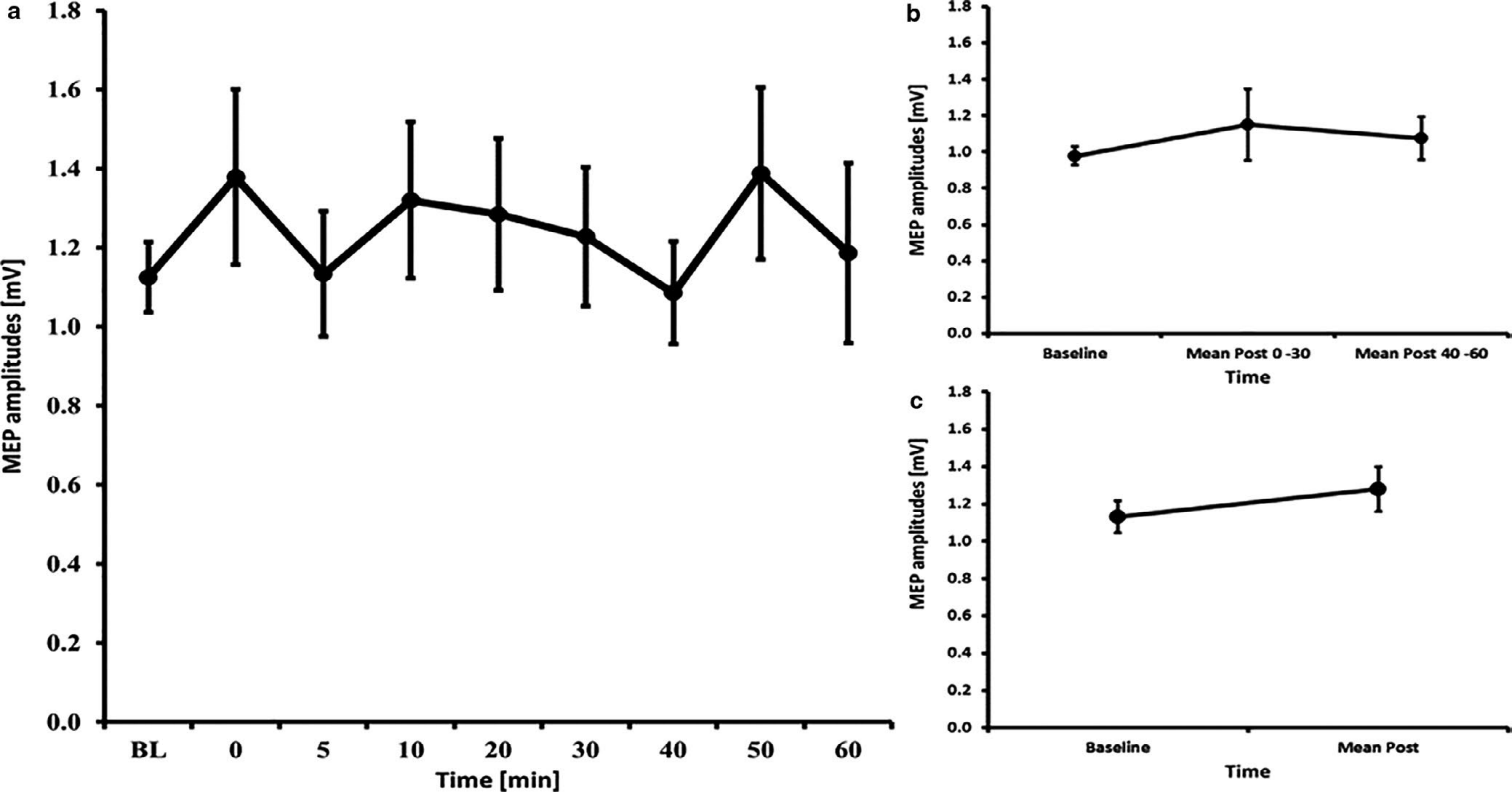
MEP courses for the 13 minutes high frequency tRNS experiments A) course of all time bins B) course of the early and late epoch time bins C) course of the post mean MEP values. Error bars refer to the standard error of the mean.

The RM‐ANOVA for all time bins revealed no significant effect of TIMECOURSE (*F*_(3.37, 47.19)_ = 1.091, *p* = .367). The RM‐ANOVA subdivided into early and late epochs revealed no significant effect of TIMECOURSE (*F*_(1.19, 16.72)_ = 0.558, *p* = .496) and paired‐samples *t*‐tests comparing baseline to mean post‐MEP values also revealed no significant differences (t(14) = 0.78, *p* = .449) (see Figure 5). The RM‐ANOVA for the I/O curves revealed a significant effect of INTENSITY (*F*_(1.32, 18.49)_ = 43.669, *p* < .001, but no effects of TIME (*F*_(1, 14)_ = 1.484, *p* = .243) or INTENSITY X TIME interaction (*F*_(1.27, 17.76)_ = 0.514, *p* = .525) (see Table 2). The RM‐ANOVA for SICI/ICF showed a main effect of ISI (*F*_(5, 70)_ = 29.660, *p* < .001), but no effect of TIME (*F*_(1, 14)_ = 1.202, *p* = .291) and of ISI X TIME interaction stimulation (*F*_(2.68, 37.48)_ = 1.543, *p* = .222) (see Table 3).

### 3.8 hf‐tRNS – 20 minutes

**Figure 6:**
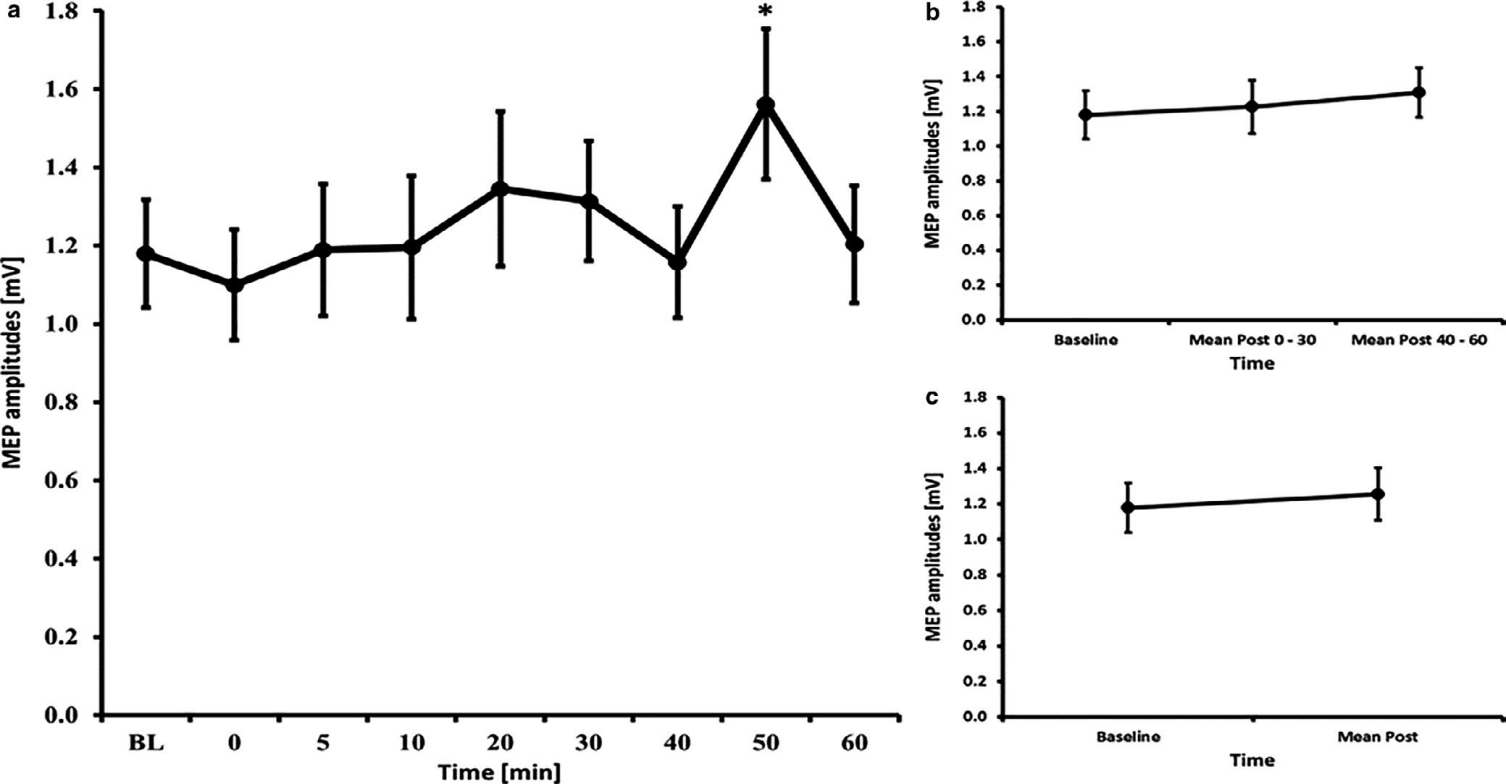
MEP courses for the 20 minutes high frequency tRNS experiments A) course of all time bins B) course of the early and late epoch time bins C) course of the post mean MEP values. Error bars refer to the standard error of the mean (*p<0.05).

For one subject we had one data point missing at 30 min poststimulation that was imputed using the mean of the 20 min and 40 min value of this subject. The RM‐ANOVA for all time bins revealed a main effect of TIMECOURSE (*F*_(8, 112)_ = 2.562, *p* = .013). LSD tests revealed significant higher MEP amplitudes compared to baseline at 50 min (*p* = .025) after stimulation, but not at all other time bins (all other time bins *p* ≥ .210). The RM‐ANOVA subdivided into early and late epochs revealed no significant effect of TIMECOURSE (*F*_(1.19, 16.62)_ = 0.888, *p* = .378) and paired‐samples *t*‐tests comparing baseline to mean post‐MEP values also revealed no significant differences (t(14)=0.70, *p* = .496) (see Figure 6). The RM‐ANOVA for the I/O curves revealed a significant effect of INTENSITY (*F*_(1.10, 15.45)_ = 28.983, *p* < .001), but no effects of TIME (*F*_(1, 14)_ = 1.500, *p* = .241) or INTENSITY X TIME interaction (*F*_(1.11, 15.50)_ = 0.979, *p* = .347) (see Table 2). The RM‐ANOVA for SICI/ICF showed a main effect of ISI (*F*_(1.89, 26.40)_ = 17.806, *p* < .001), but no effects of TIME (*F*_(1, 14)_ = 0.108, *p* = .747) or ISI X TIME interaction (*F*_(2.86, 40.06)_ = 0.614, *p* = .602) (see Table 3).

## 4 DISCUSSION

The aim of this study was to compare the efficacy of anodal tDCS and hf‐tRNS and to investigate the impact of the stimulation duration on after‐effects in motor cortical excitability of the stimulated M1. In our cohort, we were able to show an expected significant increase in motor cortical excitability only in the case of hf‐tRNS for 7 min duration and for 20 min duration, respectively. By comparison, there was a longer lasting and more significant increase in the MEP amplitude in the case of 7 min hf‐tRNS that began 20 min after the stimulation and remained significant until 60 min after the stimulation. In contrast, in the case of 20 min hf‐tRNS significant after‐effects were only observed at 50 min after the stimulation and only for this time bin. In contrast, in all tDCS experiments we were not able to show a long‐lasting after effect on the group level following either of the applied stimulation durations. A similar pattern was already described previously in a cohort of 15 subjects where 1 mA current intensity was applied both in the case of anodal tDCS and tRNS for a set duration of 10 min (Inukai et al., 2016). Here, significant increases in poststimulation MEP amplitudes were also observed only following tRNS but not in the case of tDCS compared to sham stimulation (Inukai et al., 2016). Compared to MEP magnitudes at baseline tDCS induced a significant increase in MEP amplitudes just at 20 min after stimulation whereas tRNS induced a significant increase already directly poststimulation that remained significant for 20 min after the stimulation (Inukai et al., 2016). In contrast to these findings, another foregoing study comparing five different transcranial electric current conditions (Sham, 1 mA and 2 mA anodal tDCS, 2 mA tRNS with no DC offset and 2 mA tRNS with 1 mA DC offset; set stimulation duration 10 min) showed an increase in cortical excitability following stimulation in the case of 1 mA and 2 mA anodal tDCS stimulation and 2 mA tRNS with 1 mA DC offset—however, not for 2 mA tRNS with no DC offset (Ho et al., 2015). In this context Moliadze et al., (2014) compared tDCS’ and tRNS’ efficacy using a set intensity of 1 mA and a set stimulation duration of 10 min in a cohort of 12 subjects. As a main result, the authors described significant MEP magnitude increases for both stimulation methods, while the MEP amplitude increase was strongest in the tRNS group. In the most recent meta‐analysis the application of anodal tDCS at current intensities of < 1 mA with durations > 10 min appeared to produce larger effects than higher intensities with either < 10 min or > 10 min stimulation. By comparison, all tRNS studies reviewed in this meta‐analysis demonstrated significant increases in cortical excitability regardless of specific current intensities or stimulation durations (Dissanayaka et al., 2017). This overview illustrates that our study can be viewed as a further contribution to a growing number of studies reporting on variable after‐effects in the case of anodal tDCS and—by comparison—relatively significant after‐effects following tRNS. Additionally, however, our design allowed us to gather novel experimental evidence that in the case of tRNS, a shorter stimulation duration might be associated with inducing more significant after‐effects.

This potential association of inducible after‐effects and stimulation duration was already proposed based on earlier findings (Nitsche and Paulus, 2000). Bashir et al. showed in a cohort of 32 healthy subjects that that anodal tDCS both for 10 min and 20 min with 1 mA current intensity increases motor cortical excitability, while 5 min stimulation duration failed to do so (Bashir et al., 2019). Further contributing to our understanding of the potential impact of stimulation duration, Monte‐Silva et al. reported in a cohort of 15 subjects that stimulation with anodal tDCS for 26 min resulted in a significant inhibition of the MEP amplitude after the stimulation that remained detectable for 120 min (Monte‐Silva et al., 2013). In the case of tRNS, Chaieb et al. showed in two experiments with 10 and 12 participants, respectively, that a minimum of 5 min tRNS stimulation with 1 mA was necessary to induce a significant increase in motor cortical excitability (Chaieb et al., 2011). While the stimulation durations of our tDCS paradigm do not provide novel insights beyond the heterogeneous tDCS data available for the selected stimulation durations (7 min, 13 min, and 20 min), our tRNS findings can be used to extend the conclusions of previous studies; namely that they indicate that shorter durations of tRNS, rather than longer tRNS interventions, might prove effective in inducing significant excitability increases, rather than an intermediate or longer durations of 13 min or 20 min.

Our analyses of the I/O curves and SICI‐ICF did neither show any differences between baseline and following the respective stimulation paradigms, nor for the different selected stimulation durations. Again, this extends previous observations by Ho et al. who comparatively investigated five different stimulation protocols (Sham, 1 mA and 2 mA anodal tDCS, 2 mA tRNS with no DC offset, and 2 mA tRNS with 1 mA DC offset; set stimulation duration 10 min) in 15 participants and did not observe differences between SICI/ICF at baseline and 15 min poststimulation (Ho et al., 2015). While this contextualizes our findings with previous research, our observation of nil changes with respect to SICI and ICF could also be a consequence of our methodological approach. In a recent meta‐analysis by Bibiani et al. examining after‐effects of anodal tDCS on intracortical excitability and specifically on SICI and ICF the authors observed a significant reduction of SICI parameters for ISIs of 2 ms, 3 ms, and 5 ms and a large and significant effect size in favor of an increase in ICF (Biabani et al., 2018). In contrast to our experimental approach, the measurements in the studies included into this meta‐analysis were usually performed immediately after the tDCS stimulation—while we measured SICI and ICF parameters at about 10 to 15 min following stimulation. This approach was also adopted by several other authors (Ho et al., 2015; Strube et al., 2016). Concerning I/O recruitment curves previous research also employing 13 min anodal tDCS showed a significant increase of MEP magnitudes for 110% RMT and 130% RMT but no effect for 90% RMT in a defined subgroup of tDCS responders (i.e., showing an expected increase of MEP magnitudes following tDCS) (Strube et al., 2016). Finally, with respect to tRNS, Terney et al. showed in a group of 10 subjects that tRNS applied with 1 mA for 10 min did not affect recruitment curves but observed a significant increase of ICF (12 and 15 ms) at 15 min following stimulation (Terney et al., 2008). It should be noted here, however, that their protocol differed from ours as stimulus intensities of 110%, 130%, and 150% RMT were applied for the recruitment curve and 80% of active motor threshold (AMT) were employed for the conditioning pulse of the SICI/ICF measurements (Terney et al., 2008). In Summary, our sample size might be too small to show significant after‐effects for I/O curve, or that our recording of the poststimulation parameters was too late after the stimulation to show significant after‐effects.

While our study thereby stands in line with some foregoing tDCS and tRNS findings, the generalizability of our findings is mitigated as we were not able to establish any expected facilitatory after‐effect of anodal tDCS on motor cortical excitability. This may be explained by the previously discussed inter‐subject variability (Wiethoff et al., 2014; López‐Alonso et al., 2014; Li et al., 2015; Strube et al., 2016; Fonteneau et al., 2019; Guerra et al., 2020) and is in line with other foregoing experiments reporting negative results following anodal tDCS, but may be also a consequence of our moderate sample‐size. Other limitations include that some sessions were performed only with one day break and thus carry‐over effects may have influenced the results. Concerning tRNS most previous studies were performed with 5 min and 10 min duration, respectively, which poses difficulties in the comparability to our study with 7 min duration. However, we decided for a 7 min interval to have a better adjustment to the 7 min tDCS duration. For further investigations, it would be interesting to compare 5 min and 10 min tRNS with 7 min tRNS. In addition and concerning the paired‐pulse protocols we did not adjust 1 mV single‐pulse MEP before and after the interventions to avoid changes due to single MEP changes. However, this might have resulted in less discernible after‐effects. Finally, we had no sham condition that may have allowed differentiating subtle effects of the non‐significant stimulation protocols. Finally, not all of the tests following the RM‐ANOVAs would survive correction for multiple comparisons. Specific strengths of our study are, however, that our sample size was within the range of comparable previous studies. Additionally, this is the first study systematically comparing the modifiable factor “stimulation duration” for two different stimulation paradigms (anodal tDCS versus. hf‐tRNS) using an elaborated within‐subject design.

## 5 CONCLUSIONS

In summary, this is the first systematic and randomized study to investigate the influence of stimulation duration on anodal tDCS and hf‐tRNS induced motor cortical after‐effects in a within‐subject design. First, the overall effects of the interventions were below our expectations. Second, our findings reconfirmed the current view that hf‐tRNS might be more effective than anodal tDCS in inducing significant and long‐lasting changes in motor cortical excitability. Further, our findings indicate that shorter stimulation durations of 7 min might be superior compared to longer stimulation durations of 13 min and 20 min, respectively. To further evaluate these findings and to address the issue of inter‐individual variability in the context of stimulation duration as well as in the combination of current intensity, further randomized studies are needed.

## 6 CONFLICT OF INTEREST STATEMENT

Alkomiet Hasan has received a paid speakership from Janssen‐Cilag, Otsuka, and Lundbeck. He was a member of an advisory board of Roche, Janssen‐Cilag, Otsuka, and Lundbeck. Wolfgang Strube has received a speaker's honorarium from Mag&More GmbH. Jan Haeckert, Christoph Lasser, and Benjamin Pross declare no conflicts of interest.

## AUTHOR CONTRIBUTION

Jan Haeckert performed experimental procedures, data analysis, and statistical analysis. He wrote the paper and drafted the tables and figures. Christoph Lasser performed experimental procedures, data analysis, and statistical analysis. Benjamin Pross revised the paper. Alkomiet Hasan invented the study, supervised the experimental procedure, revised the data analysis, statistical analysis, and the paper, tables, and figures. Wolfgang Strube revised the paper, the data and statistical analysis, the tables and figures.
